# Ten simple rules for giving an effective academic job talk

**DOI:** 10.1371/journal.pcbi.1007163

**Published:** 2019-07-25

**Authors:** Shayna A. Sura, Lauren L. Smith, Monique R. Ambrose, C. Eduardo Guerra Amorim, Annabel C. Beichman, Ana C. R. Gomez, Mark Juhn, Gaurav S. Kandlikar, Julie S. Miller, Jazlyn Mooney, Riley O. Mummah, Kirk E. Lohmueller, James O. Lloyd-Smith

**Affiliations:** 1 Department of Ecology and Evolutionary Biology, University of California Los Angeles, Los Angeles, California, United States of America; 2 Department of Human Genetics, University of California Los Angeles, Los Angeles, California, United States of America; Whitehead Institute for Biomedical Research, UNITED STATES

## Introduction

You’ve finally completed your dissertation research and have your PhD in hand—yay! Maybe you’re also in the middle of a postdoctoral position. If you’re reading this article, chances are you are actively searching for and applying for faculty positions. (Check out reference [1] if you’re early in the application process and [2] for additional advice!) Unfortunately, many graduate students and postdocs are not taught the skills necessary for acquiring a faculty position after passing the “looks good on paper” part of the application and securing an on-campus interview. One of the last crucial steps in earning a faculty position is your academic job talk. No matter how great of a scientist you are, if you cannot give a compelling job talk, chances are low that you will be hired. Yet many candidates receive little guidance on how to ace this unique and vital test.

To help address this gap, we have put together these ten simple rules that will help you give an effective job talk. To be clear, these are rules developed for the academic job talk in a research-heavy department, which is typically in a seminar format. These rules are not targeted toward other formats such as chalk talks or teaching demonstrations, although some pointers may still apply. We are a group primarily composed of University of California, Los Angeles (UCLA) faculty, postdocs, and graduate students who participated in two recent job searches in the Ecology and Evolutionary Biology Department. We evaluated ten job talks over the span of 2 months and discussed their strengths and weaknesses in a weekly seminar course. These ten rules are based on our discussions of what worked (and what didn’t) across the variety of job talks we observed, as well as our various experiences on the job market and search committees over the years.

## Rule 1: Know your audience

As with any seminar or presentation, when preparing your job talk, you want to target your specific audience. Therefore, you need to consider the background knowledge and interests of the audience members. Learn as much as you can about the position and what institutional needs the position is meant to address within the department and broader university. If you’re applying for a position within a specific department, what is the scope of the research in that department? Does it have a mission statement? Are any strategic aims or future plans publicly available? Does the department work closely with other academic units on campus, and does the position you’ve applied for have any formal ties to other units? To answer some of these questions, you should read the job ad closely, read about the current faculty’s research, and look through the department’s web page (see also Rule 7 [Understand your potential new workplace] and 8 [Understand your new colleagues] from reference [3]). If you’re lucky enough to have network connections to the department, use them now to get insights before you visit. We also recommend that after you receive an invitation to interview, you consider setting up a phone call with the chair of the search committee to inquire about the job and ask any specific questions you have regarding the job or department. In particular, it is a good idea to ask what the search committee is looking for—it may have been a long time since the job ad was released, and the search committee’s focus may have shifted from what was initially stated. We recommend a phone conversation as opposed to an emailed list of questions because it saves time; also, people are often more candid and may provide more useful insights over the phone. Depending on when your job talk occurs during your interview schedule, you might even make small changes to customize your talk based on interviews and meetings with department members prior to your talk.

## Rule 2: Sell yourself

The faculty and search committee are trying to choose the candidate they’ll be most excited to have as a new colleague, so you need to showcase the reasons you’re their best choice! It is smart to include an explicit introduction about yourself—i.e., the kind of science you do, your grand aims, and your approach to research. You want to communicate your identity as a researcher and, if appropriate given your career stage and research plans, how this differentiates you from your mentors (reference [4] is an excellent resource).

You also want to convey other traits as a scientist and potential colleague. Reflect on the qualities that make you an exceptional researcher (creative, persistent, thoughtful, rigorous, multidisciplinary, etc.), as well as the specific traits that your audience will be looking for, and try to demonstrate them subtly to the audience over the course of the talk via examples in your work. Consider ways to demonstrate your fundamental strengths as a scientist, such as the ability to question your methods and results to pursue deeper and more robust conclusions. If you have any particular successes on your record, such as big grants or markers of professional stature, don’t be shy about mentioning them (but don’t brag!). Having your publication citations and/or grants listed in smaller text at the bottom of corresponding slides is one way to show your accomplishments without explicitly mentioning them. Finally, you can casually highlight additional non-research skills (e.g., mentoring, outreach, collaborations) throughout your talk. For example, give credit to an excellent mentee who contributed to the data collection or to a gifted collaborator who added a component to your study. Your application materials likely included many of these things, but if you can find ways to incorporate them in your talk, a broader audience can see the full package of who you are.

Keep in mind Rule 1 (Know your audience) when deciding how best to showcase yourself, as different disciplines and subfields may vary in their perceptions of what makes a good scientist. For example, disciplines may vary in their appreciation for deep thought into specific mechanisms and experimental designs versus mathematical elegance and rigor. Others may prize applied over fundamental research or vice versa. This may be especially challenging if your research is interdisciplinary, so make sure to investigate what factors are valued most highly by the decision makers in the audience for your talk so you can design your talk to emphasize those aspects of your work.

## Rule 3: Impress the in-crowd…

Likely there will be people in the audience who work in the same field as you. Make sure to impress these experts with your knowledge and convince them you are worthy of being their colleague. You want to show them you have the sophistication and skills necessary to tackle advanced problems. Therefore, it’s a good idea to do at least one “deep dive” during your talk in which you include one or two “muscle-flexing” slides. By this we mean slides with technical content that the general audience member may not be able to fully understand but for which you can flex your intellectual muscles and showcase your skills. Importantly, do not bluff or bluster in this section—a technical error in your deep dive would be fatal.

These deep dives shouldn’t be long, or you risk losing most of your audience. However, a glimpse into the more advanced aspects of your work will convey that you’re able to play in the big leagues in your field. Just make sure to reengage your audience after this show of prowess, ideally providing a big-picture summary of what you’ve just shown.

## Rule 4: … but also appeal to the out-crowd

In addition to impressing the specialists in the audience, you want to make sure the people who work outside your discipline are able to follow and enjoy your presentation. When preparing your talk, consider how you can present and frame the material so that even audience members from far-flung disciplines are engaged and can appreciate the broader relevance of your presentation. Be attuned to the breadth of the department you’re visiting, as this can present various communication challenges. The diverse interests of faculty in a broad department (e.g., biology) can make it difficult to make your research program appealing to everyone. However, it can also be difficult communicating to a more focused department (e.g., molecular genetics) if your research is not exactly in line with what everyone else does. It helps to summarize the important findings of your research as you present them, in addition to their implications and why they are exciting, in case not everyone followed the technical aspects of your results. You can also make it easier for audience members from other fields to follow your talk by avoiding excess jargon and keeping your messages clear.

Emphasize the themes in your work that relate to the job and department you’re interviewing for. If applicable and appropriate, it can help to subtly highlight connections between your research to research of other members of the department who have different specialties. But be careful not to overdo this, as it can become distracting.

## Rule 5: Play the hand you’ve got to optimal effect

Strategic choice of topics to include in your talk from among your entire research portfolio is critical for giving an effective and memorable job talk. Depending upon what career stage you are in (just finished PhD, postdoc, assistant professor, etc.), you may have a smaller or larger research portfolio. For an hour-long job talk, it is unlikely you will be able to effectively discuss everything you have ever done. And that’s okay, because that is what a CV is for!

**Fig 1:**
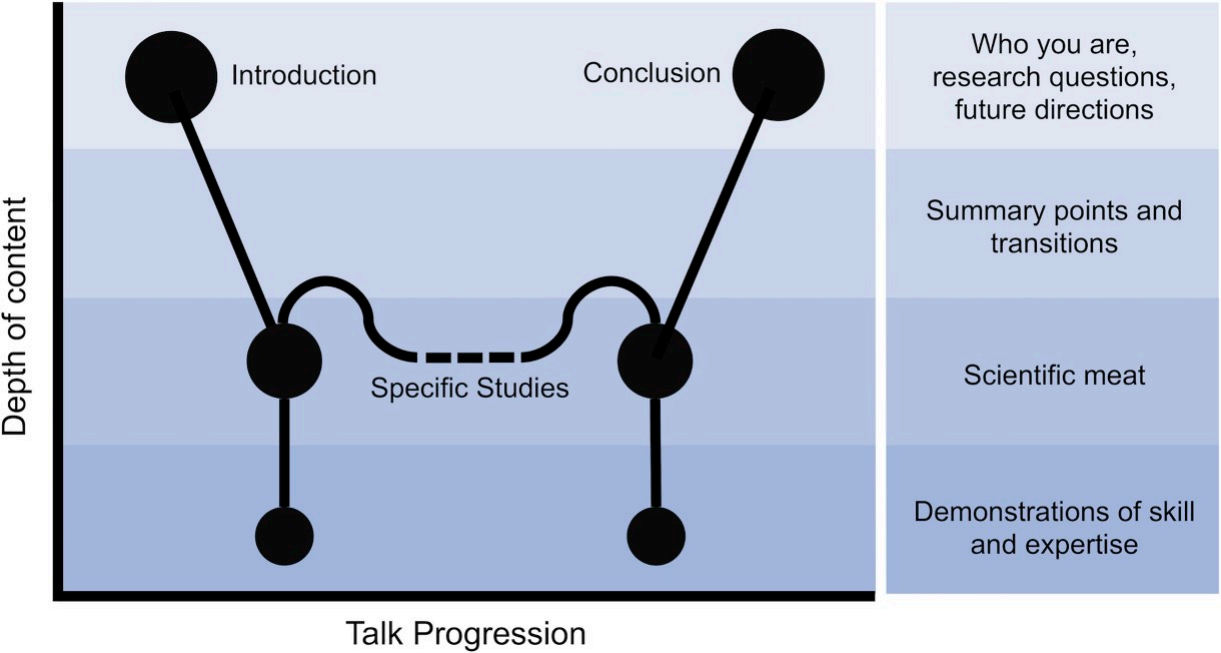
Depth of content throughout the talk. You want to start broad during the introduction to get everyone on board and then go into more depth on a few specific studies, including some “deep dives” to show off expert knowledge. Finally, you want to conclude your talk on a broad scale similar to your introduction. The dashed lines indicate flexibility in how many specific studies you incorporate into your talk, based upon your own research portfolio.

For your job talk, you need to assess your portfolio of published work, unpublished but completed work, and ongoing projects to determine which projects showcase your work most effectively and best match what the department is looking for in a future colleague. The most effective talk structures we observed were ones that focused on 2–3 research studies and that combined higher-level information with a few “deep dives” into the nitty gritty of a particular study (Fig 1). This talk structure will help you satisfy Rules 3 and 4 above, which discuss how you want your whole audience to understand and appreciate your talk, while also presenting the “meat” of your research and impressing those most familiar with your field. If you feel that this design doesn’t convey the breadth or quantity of your productivity, consider adding a slide or two on the conceptual structure of your full research program in which you can show (with all your best citations) how all the pieces fit together.

In addition to presenting on your past and ongoing research, you need to clearly articulate your plan for your future research program. Tell the audience (and your potential future colleagues!) about your vision for your research lab both in the immediate future (next couple of years) and in the long term (5–10 years from now). This should also help differentiate you and your research from your previous mentors and their research programs. A critical part of establishing and maintaining a research program is your ability to generate funding. If you have already secured funding for your future research plans or you have a track record of successfully acquiring funding, then this is a great opportunity to bring this to your audience’s attention. If you don’t have independent funding yet, you can still demonstrate your awareness of the funding landscape and which funding opportunities are likely to support your research program. For example, in your future directions section, you might briefly touch on how one (or more) of your research questions aligns well with promising funding opportunities in your field, such as open research grants.

In organizing the structure of your talk and your transitions between topics, strive for a cohesive narrative that will make your talk more enjoyable to follow and easier to recall afterwards. What’s the progression of your research? How did one study lead to the next, and what shaped your decisions about how to proceed? What ideas do you have for future research at this new job? Telling a story is always a great way to keep your audience engaged and makes your science more memorable.

## Rule 6: Give a good talk

A classic early paper in this series [5] provides ten useful rules for giving a good presentation. Read it! Showing you are a competent oral communicator is a vital component of giving an academic job talk. In addition to the universal suggestions from [5] (such as practicing for fluidity without over-rehearsing, making eye contact with the audience, and being enthusiastic and excited about your work), there are a few other pointers to bear in mind for a job talk. First, be aware that your job talk will be judged as an indicator of your ability to teach. Teaching is a crucial element of most academic jobs, but interview schedules often don’t allow time to address it explicitly, so this doubles your incentive to give a clear and engaging presentation. Bonus points if you are able to expand people’s understanding of technical aspects of your work—for instance, with a lucid explanation of your deep dive. Second, the job talk is a direct measure of your ability to sell your work and to act as an ambassador for the department in your future speaking engagements. Third, Rule 4 from [5] is “Make the take-home message persistent,” and this is a particular priority in the swirl of an academic search in which four or five candidates may visit over the span of a few weeks. We found that a strong thematic structure, including outline and summary slides, was an effective way to emphasize and reiterate your key points and make them memorable for the audience.

Our next three pointers are more pragmatic, but they are still useful to consider. First, be sure to ask for guidance on talk length if you’re unsure. For an hour-long seminar, the actual presentation length is typically 45–50 minutes, allowing for the fact that your host may burn precious minutes introducing you, and being certain to leave time for questions. Second, you should also make sure you understand the audiovisual equipment setup in the room where you are giving your presentation. If there isn’t seminar preparation time on your schedule, ask for it! This way, you can ensure your presentation is loaded properly, your presentation slides appear how you expect, and you are able to navigate through them without glitches. It is a good idea to save your presentation in multiple formats in case you encounter compatibility issues with the primary format (e.g., if your presentation is in PowerPoint, also save a PDF backup version). Third, don’t give your presentation while hungry. You want to exude energy and confidence, which may be difficult if you give a seminar later in the afternoon after many meetings and haven’t eaten since lunch—so take note of your schedule and, if necessary, bring a snack to revive your energy levels before your talk.

The pragmatic pointers we mentioned are great for planning ahead, but overall, you should be adaptable. Problems can arise unexpectedly, and it’s possible you’ll be delayed by interruptions or a lengthy introduction. Do your best to not get flustered, to handle yourself with grace, and to end your talk on time. Make a note of places in your talk where you can go into greater depth if you’re running ahead of schedule or places (particularly toward the end) where you can skim over the details more quickly if you’re behind schedule.

## Rule 7: Be kind to your audience’s eyes

Your slides should enhance your presentation, not distract from what you are saying. Make sure your slide aesthetics are appealing to the audience. Your slides should be clear and concise, without too much text. When you have text-heavy slides, you lose some proportion of your audience’s attention while they read the text instead of listening to your words. So only display text that emphasizes the key points you will say out loud. Also, since the figures and images you present are especially important, you will want to construct figures specifically for your slides, keeping in mind that formatting for a presentation is typically different from formatting for a published paper. Refer to Box 1 for additional advice on qualities of good slides and common mistakes to avoid. You should also check out [5,6] for additional advice, noting that the rules in [6] are not specific to figures for presentations.

**Box 1. Qualities of good slides versus slide qualities to be avoided**

Slide qualities to aim for:

- Good content:
  ○ Minimal text.
  ○ Figures that are readable and easily understood.
  ○ Figures created specifically for talks (rather than pulled directly from a paper). Talk figures are generally simpler than figure panels from a paper, with fewer items per plot, a focus on the key points, larger labels and axes, etc. Avoid having to tell your audience to ignore parts of the figure by remaking the figure without extraneous information.
  ○ If you have a complicated figure, you can animate your slides to build up the complexity as you explain it to the audience. For example, you can start by showing only a very simple plot and then layer on additional pieces of information as you explain them.
- Good design:
  ○ Clean background.
  ○ Consistent design throughout the talk.
  ○ Color-blind-friendly color palettes or alternative ways to distinguish differences on figures besides just color (e.g., using dotted versus solid lines to represent different measures in a plot).
  ○ Simple visual markers (silhouettes or clip art) that link ideas across slides and jog your audience’s memory (e.g., a human silhouette next to parameters estimated from human data and a mouse silhouette next to data estimated from mice).

Slide qualities to avoid:

- Too much text.
- Text that’s too small to read or overlaid on an image so that it’s not legible.
- Busy background (e.g., photograph) that distracts from the text and/or figures you’re showing on the slide.
- Figures with no or unreadable axis labels.
- Poor color combinations, including combinations that are difficult for color-blind viewers to make out (e.g., red/green, blue/green).
- Visual markers that don’t convey any meaningful information, such as changing fonts and background colors. Even minor inconsistencies are distracting and convey a lack of attention to detail.

## Rule 8: Embody the future

Remember that you are the exciting next generation of scientists! Make sure to share your enthusiasm and your fresh ideas for research. Emphasize how your work is new and innovative, whether by showing new solutions to old problems or by describing ways to approach problems that have only recently been recognized. If appropriate, highlight how you will harness the latest technologies and methodological developments to advance your research. This will get the audience thinking about applications to their own research programs and how you’d be a valuable colleague to have around.

You can also emphasize other forward-looking traits you would bring to the job. Maybe you have developed a new online resource or are using a new mentoring or teaching style that helps make research more broadly accessible for students. Find ways to showcase how you are moving science forward and how you’ll be a dynamic force for years to come.

## Rule 9: Don’t blow it in the question-and-answer session

You’re almost done with your job talk, so don’t blow it during the question-and-answer (Q&A) session! You want to leave your audience with the best final impression and show that you can think and speak clearly in unscripted moments.

Here are some tips for a strong finish. When someone asks you a question, it can be helpful to paraphrase the question before beginning your answer. This gives you some extra time to compose your own thoughts and make sure you understood the question and ensures the rest of the audience hears the question. Regarding your actual responses, one cardinal rule is to never bluff. If you don’t know the answer, you can say so, but then show how you would think through the question, or relate it to something you have done or know about. If somebody voices a fair criticism, then acknowledge it and discuss approaches to addressing it. If you can, convey enthusiasm in this situation—if it’s truly an idea you’ve never considered, then treat this as an exciting and valuable scientific exchange, not an oral exam you are failing.

Remember that your audience likely includes people from outside your area of expertise, so it is possible you will get questions that seem to have missed key ideas from your talk. As with all questions, make sure you understand what the questioner is asking, and then take advantage of the opportunity to address any misunderstandings in a respectful, productive way. This is a great chance to demonstrate your ability to explain concepts clearly and concisely.

If there are predictable follow-up questions to your presentation, it can be helpful to have a few extra slides prepared. For example, if you presented a mathematical model using a schematic diagram, you may want to have a backup slide that shows the actual equations in case someone asks for more detail. If there is an extra data set or analysis that you’d love to include but just don’t have the time, then a spare slide or two might enable you to deliver a home-run response if you get asked the right question.

Finally, remember to handle yourself with grace during the Q&A session. Be poised, calm, and respectful, and demonstrate your intellectual maturity—all of these are qualities people admire and are seeking in a future colleague. Another past article in this series gives rules for building your scientific reputation [7]; Rules 1, 2, and 3 are useful during both the Q&A session and the whole interview process! Which brings us to Rule 10.

## Rule 10: Be professional

Throughout this whole process, remember you are asking the host department to hire you as a (hopefully) long-term colleague in a small, tight-knit unit. Therefore, it is important to present a good image of yourself. You should dress appropriately for your job talk (i.e., not too casually). Even if you end up being a bit overdressed, it is better to leave that impression rather than showing up underdressed and being remembered as not having taken the job talk seriously. Be conscious of your body language and use of slang throughout your job talk and in any interactions you have during your visit. Humor can be a wonderful way to humanize and enliven your talk, but don’t overdo it, and steer well clear of anything potentially offensive. While you are answering questions, or if you happen to be interrupted during your talk, remember to show yourself in the best light by being polite and calm, even if an audience member is being confrontational or rude.

You are an amazing and productive scientist (you wouldn’t have been invited to give a job talk if you weren’t!), but it’s important to be clear about your specific contributions to the various research projects you present, particularly when the research is part of a big collaboration. It’s essential to acknowledge your collaborators, especially junior mentees. This shows your audience that you are ready to mentor undergraduates, graduates, postdocs, etc., and most importantly, that you do not take collaborators’ contributions for granted or claim them as your own. It’s also good practice to acknowledge relevant previous work that your research and ideas are building upon, as you never know who is in your audience, and you don’t want anyone to feel you are uninformed about or taking credit for this prior research. Again, you’re asking to be hired into an academic family, and you want your new family members to be comfortable and excited about pursuing new research opportunities with you.

Finally, it is a nice touch to write thank-you notes after your visit (but see Rule 10 from [3] for an alternative opinion). These notes can be sent by email within a few days after the end of your job interview. How many you send is up to you, but we suggest sending follow-up notes to at least the search chair and the other key players in your interview visit. And don’t forget about all the people who helped coordinate the logistical details for your visit!

In summary, the academic job talk is unlike most other seminars in its goals, context, and aspects of its execution. We have outlined some rules to help you put your best face forward in the job market (and to help all of us get the most out of the job search experience). There are additional resources online (e.g., [8] and [9] as two examples), and people should glean whatever insights they can from these sources. So do your preparation, nail the talk, and go get that job!

## References

[1] Tomaska L, Nosek J (2018) Ten simple rules for writing a cover letter to accompany a job application for an academic position. PLoS Comput Biol 14(5): e1006132 10.1371/journal.pcbi.1006132 29851981 PMC5978783

[2] Bourne PE (2011) Ten Simple Rules for Getting Ahead as a Computational Biologist in Academia. PLoS Comput Biol 7(1): e1002001 10.1371/journal.pcbi.1002001 21253560 PMC3017106

[3] Bourne PE (2014) Ten Simple Rules for Approaching a New Job. PLoS Comput Biol 10(6): e1003660 10.1371/journal.pcbi.1003660 24967974 PMC4072506

[4] Botham CM, Arribere JA, Brubaker SW, Beier KT (2017) Ten simple rules for writing a career development award proposal. PLoS Comput Biol 13(12): e1005863 10.1371/journal.pcbi.1005863 29240828 PMC5730102

[5] Bourne PE (2007) Ten simple rules for making good oral presentations. PLoS Comput Biol 3(4): e77 10.1371/journal.pcbi.0030077 17500596 PMC1857815

[6] Rougier NP, Droettboom M, Bourne PE (2014) Ten Simple Rules for Better Figures. PLoS Comput Biol 10(9): e1003833 10.1371/journal.pcbi.1003833 25210732 PMC4161295

[7] Bourne PE, Barbour V (2011) Ten Simple Rules for Building and Maintaining a Scientific Reputation. PLoS Comput Biol 7(6): e1002108 10.1371/journal.pcbi.1002108 21738465 PMC3127799

[8] Reis RM. Giving a job talk in the sciences. 30 March 2011 [cited 2019 May 15]. In: The Chronicle of Higher Education [Internet]. Available from: https://www.chronicle.com/article/Giving-a-Job-Talk-in-the/45375.

[9] Aguilar SJ. Tips for a successful job talk. 10 January 2018 [cited 2019 May 15]. In: Inside Higher Ed [Internet]. Available from https://www.insidehighered.com/advice/2018/01/10/advice-giving-effective-job-presentation-opinion.

